# Pelvic Inflammatory Disease Complicated by a Tubo-Ovarian Abscess: A Case Report and Literature Review

**DOI:** 10.7759/cureus.89230

**Published:** 2025-08-01

**Authors:** Clifford Pang, Katherine Pang, Seeduwa M Bandara, Darrick Lee

**Affiliations:** 1 Department of Medicine, University of California Los Angeles David Geffen School of Medicine, Los Angeles, USA

**Keywords:** diagnosis and treatment, infectious disease, pelvic inflammatory disease (pid), sexually transmitted diseses, tubo ovarian abscess

## Abstract

Pelvic inflammatory disease (PID) is an infection of the female upper genital tract structures involving the uterus, oviducts, ovaries, and/or other surrounding pelvic organs. Clinical diagnosis of PID is of utmost importance as there is currently no single gold standard diagnostic test. Here, we present a case of a 47-year-old woman presenting with left lower quadrant abdominal and suprapubic pain who was found to have PID complicated by tubo-ovarian abscess (TOA). This case highlights the importance of a thorough history and physical exam in the diagnosis of PID.

## Introduction

Pelvic inflammatory disease (PID) is an infection of the female upper genital tract that results in a wide spectrum of clinical presentations, including endometritis, salpingitis, oophoritis, tubo-ovarian abscess (TOA), peritonitis, and perihepatitis (Fitz-Hugh-Curtis syndrome) occurring in approximately 12% of patients with pelvic inflammatory disease [1]. In the United States, approximately 4% of females aged 18-44 reported having pelvic inflammatory disease, which accounts for approximately 90,000 outpatient visits per year [2]. The majority of pelvic inflammatory disease cases (over 85%) are caused by sexually transmitted or bacterial vaginosis-associated pathogens, and less than 15% of cases are caused by an enteric or respiratory pathogen [3]. Although there is no gold standard diagnostic test for pelvic inflammatory disease, prompt diagnosis and management of this condition are necessary due to the risk of acute and long-term consequences, including chronic pelvic pain, infertility, and ectopic pregnancy. We present a case of acute pelvic inflammatory disease complicated by tubo-ovarian abscess (TOA), which occurs in 10-35% of patients with PID [4].

## Case presentation

A 47-year-old female with a past medical history significant for recurrent diverticulitis requiring hospital admission, recurrent urinary tract infections, morbid obesity with a body mass index of 47, and premenstrual dysphoric disorder who presented to her primary care physician with two days of left lower abdominal and suprapubic pressure with burning sensations. Pertinent positives on review of systems included vaginal discharge, dysuria, chills, and nausea. She admitted to sexual intercourse a month prior to symptom onset with a partner who ultimately tested positive for chlamydia, and she took a dose of post-coital nitrofurantoin afterwards. 

In the office, her vital signs showed a temperature of 96.9 degrees Fahrenheit, blood pressure 119/82 mmHg, heart rate 98 beats per minute, respiratory rate 18 breaths per minute, pulse oxygenation 97% on room air. On physical examination she was well appearing and in no acute distress. Abdominal examination was significant for tenderness to palpation in the left lower quadrant and suprapubic region. A pelvic examination was performed and was significant for cervical motion tenderness and nonpalpable, nontender adnexa and uterus. Urine dipstick was sterile and negative for blood, nitrite, and leukocyte esterase. She was initiated on treatment for presumed pelvic inflammatory disease (PID) with intramuscular (IM) ceftriaxone 500 mg followed by a 14-day course of oral doxycycline 100 mg every 12 hours plus metronidazole 500 mg every 12 hours. Labs returned showing a new leukocytosis with white blood cell count 17.69 x10^3^/uL and neutrophil predominance. Vaginal swabs were negative for *Chlamydia trachomatis/Neisseria gonorrheae* by Polymerase Chain Reaction (PCR), *Bacterial vaginosis *screen, *Trichomonas vaginalis* antigen, and fungal culture. Human Immunodeficiency Virus (HIV)-1/2 Antigen/Antibody, Hepatitis C Virus (HCV) Antibody, and Rapid Plasma Reagin (RPR) were nonreactive (Table 1).

**Table 1:** Labs on initial presentation

Laboratory Test	Reference Range	Patient’s Values
White Blood Cell Count	4.16-9.95 x10^3^/uL	17.69 x10^3^/uL
Hemoglobin	11.6 - 15.2 g/dL	12.9 g/dL
Platelet Count	143 - 398 x10^3^/uL	383 x10^3^/uL
Creatinine	0.6 - 1.30 mg/dL	0.72 mg/dL
Aspartate Aminotransferase	13 - 62 U/L	16 U/L
Alanine Aminotransferase	8 - 70 U/L	17 U/L
Human Immunodeficiency Virus (HIV)-1/2 Antigen/Antibody Screen 4th Generation	Nonreactive	Nonreactive
Hepatitis C Virus Antibody Screen	Nonreactive	Nonreactive
Rapid Plasma Reagin with Treponema Pallidum Particle Agglutination (RPR with TP-PA)	Nonreactive	Nonreactive
Chlamydia trachomatis Polymerase Chain Reaction (PCR) vaginal swab	Negative	Negative
Neisseria gonorrheae Polymerase Chain Reaction (PCR) vaginal swab	Negative	Negative
Trichomonas vaginalis Antigen vaginal swab	Negative	Negative
Bacterial vaginosis Screen vaginal swab	Gram stain not consistent with bacterial vaginosis (Modified Nugent Score 0-3)	Gram stain not consistent with bacterial vaginosis (Modified Nugent Score 0-3)
Fungal Culture vaginal swab	Negative	Negative
Blood, urine dipstick	Negative	Negative
Nitrite, urine dipstick	Negative	Negative
Leukocyte Esterase, urine dipstick	Negative	Negative

**Figure 1:**
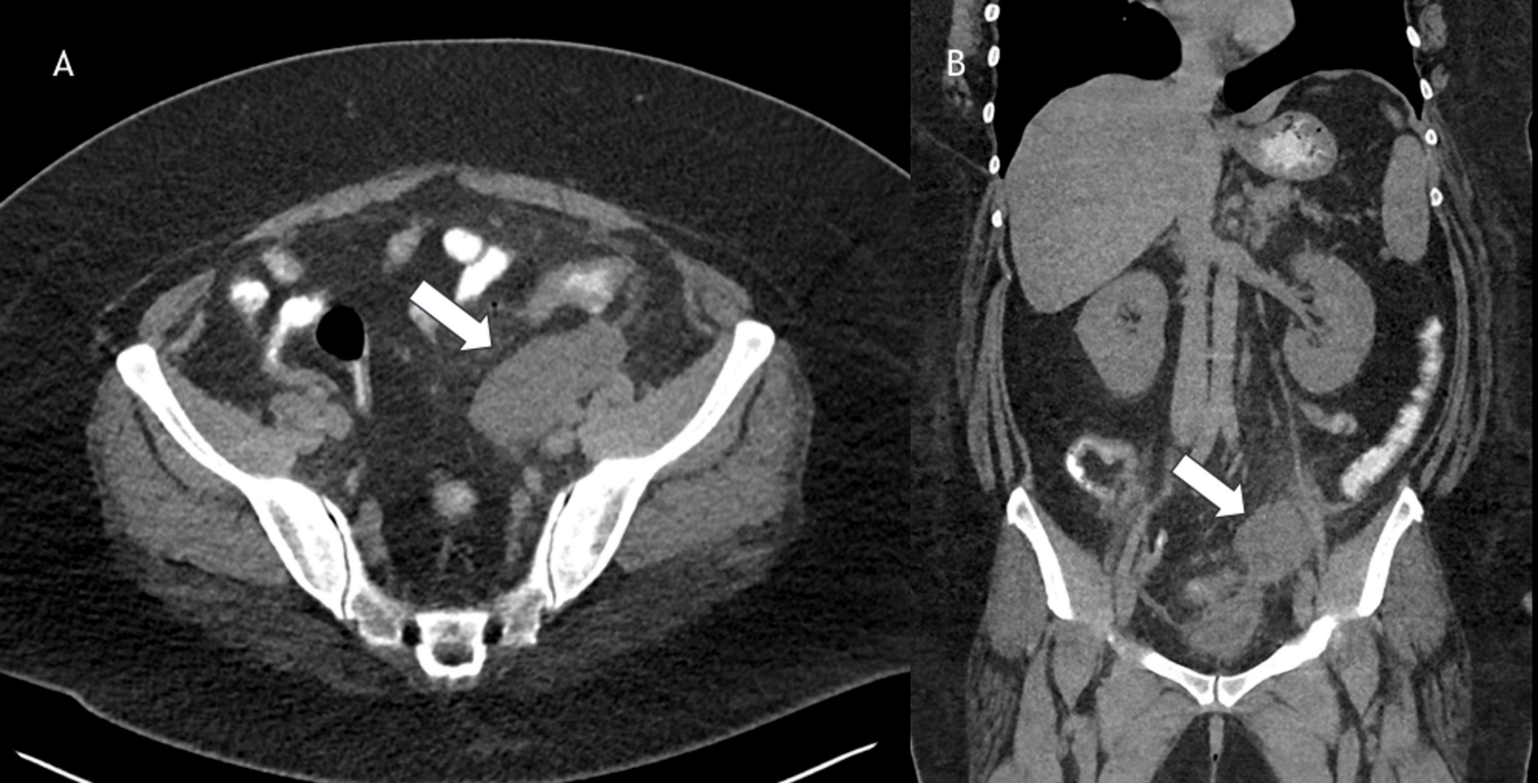
Non-contrast computed tomography (CT) showing left adnexal lesion with surrounding fat stranding (1A) Transverse view, (1B) Coronal view.

**Figure 2:**
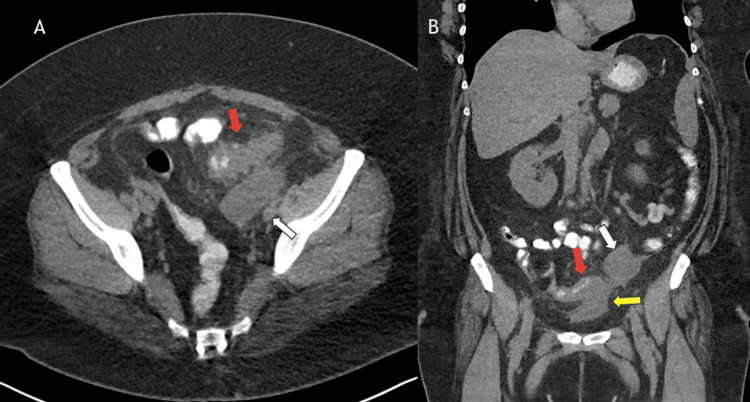
Non-contrast computed tomography (CT) a few slices lower showing all of the findings (2A) Transverse view, (2B) Coronal view White arrow: Round left adnexal lesion with surrounding inflammatory fat stranding. Red arrow: Mural thickening of the sigmoid colon with multiple inflamed diverticula, may be reactive in setting of possible tubo-ovarian abscess or suggestive of diverticulitis. Yellow arrow: Possible inflammatory thickening and continuity between the sigmoid colon and bladder (decompressed with minimal amount of urine) suggestive of colovesicular fistula.

Over the next three days, she began to worsen clinically and ultimately presented to the emergency room with worsening lower pelvic pain. In the emergency room, her vitals were stable and she was afebrile. Labs showed a white blood cell count of 13.02 x 10^3^/uL and normal lipase 26 U/L. Urinalysis with microscopy was repeated and showed 0 red blood cells/high power field, 1 white blood cell/high power field, and no bacteria (Table 2). Computed tomography (CT) of the abdomen without contrast (due to the patient's contrast allergy) showed a new left adnexal mass-like density with significant surrounding fat stranding and reactive inflammatory changes involving the adjacent sigmoid colon and bladder without evidence of diverticulitis (Figures 1, 2).

**Table 2:** Labs on hospital presentation

Laboratory Test	Reference Range	Patient’s Values
White Blood Cell Count	4.16-9.95 x10^3^/uL	13.02 x10^3^/uL
Hemoglobin	11.6 - 15.2 g/dL	12.8 g/dL
Platelet Count	143 - 398 x10^3^/uL	422 x10^3^/uL
Creatinine	0.6 - 1.30 mg/dL	0.70 mg/dL
Aspartate Aminotransferase	13 - 62 U/L	12 U/L
Alanine Aminotransferase	8 - 70 U/L	6 U/L
Lipase	13 - 69 U/L	26 U/L
Red Blood Cell/High Power Field (RBC/HPF)	0 - 2 cells/HPF	0 cells/HPF
White Blood Cell/High Power Field (WBC/HPF)	0 - 4 cells/HPF	1 cells/HPF

**Figure 3:**
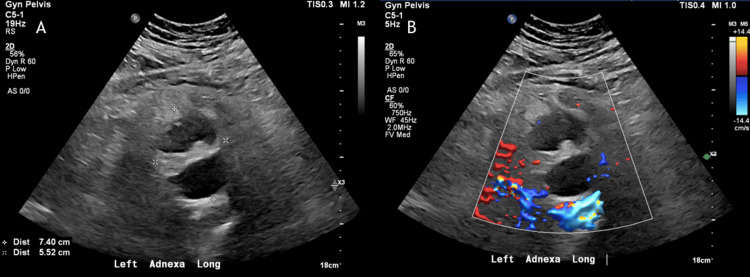
Gray-scale and doppler pelvic ultrasound demonstrating the previously seen lesion on computed tomography (CT) (1A) Gray-scale ultrasound, (1B) Doppler ultrasound Complex left adnexal lesion with multiple anechoic cystic-like lesions with trace internal echoes, thick hyperechoic septations, and surrounding hyperemia measuring up to 7.4 x 5.5 cm may reflect tubo-ovarian abscess.

A pelvic ultrasound was performed, and the radiology report read as a “complex left adnexal cyst-like lesion with suggestion of thick septations and surrounding hyperemia may reflect tubo-ovarian abscess” (Figure 3). She was admitted to the hospital, and treatment was initiated with intravenous (IV) ceftriaxone 1 g every 24 hours, intravenous (IV) doxycycline 100 mg every 12 hours, and IV metronidazole 500 mg every 12 hours. Interventional radiology was consulted for possible drainage of the tubo-ovarian abscess, but due to the patient’s contrast allergy, they were unable to visualize the fluid collection. The patient’s pain improved, and her white blood cell count downtrended and she was transitioned to oral Doxycycline 100 mg every 12 hours and Metronidazole 500 mg every 12 hours to complete a total 14-day course. 

**Figure 4:**
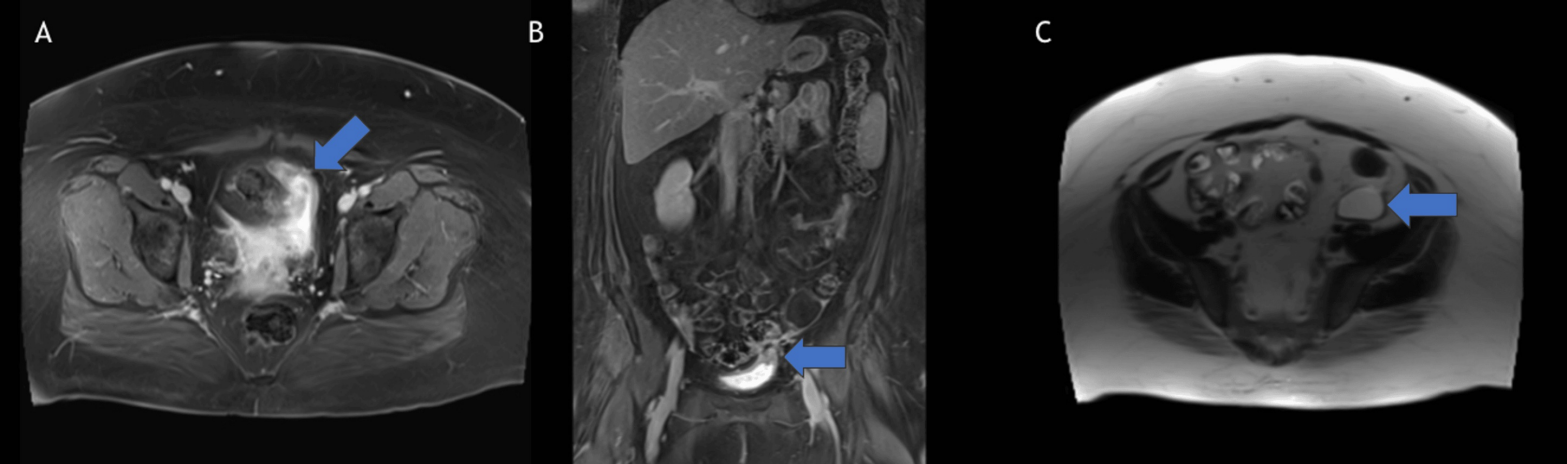
Magnetic resonance (MR) urogram February 2025 (4A) Transverse view with T1 contrast enhanced fat saturated Magnetic Resonance Imaging (MRI) demonstrating focal contact between a sigmoid diverticulum and the left bladder base, with some surrounding inflammatory change suggestive for colovesicular fistula. (4B) Coronal view with T1 contrast enhanced fat saturated Magnetic Resonance Imaging (MRI) demonstrating focal contact between a sigmoid diverticulum and the left bladder base, with some surrounding inflammatory change suggestive for colovesicular fistula. (4C) Transverse view with T2 weighted MRI demonstrating hyperintense contents of previously seen left adnexal lesion without any septations or nodularity, representing fluid and suggestive of a simple left adnexal cyst.

She recovered from acute PID but was ultimately found to have an enterovesicular fistula, thought to have developed as a complication of her recurrent diverticulitis (Figure 4). It is possible that her PID may have been caused by enteric pathogens, which represent fewer than 15% of acute PID cases [3].

## Discussion

This case illustrates the importance of a thorough clinical evaluation and low threshold to initiate empiric treatment for suspected cases of pelvic inflammatory disease (PID) as the diagnosis is made clinically. Pelvic inflammatory disease should be suspected in sexually active young females presenting with lower abdominal or pelvic pain. The cardinal presenting symptom of PID is lower abdominal pain, which is usually bilateral and can worsen with coitus or positional movements. Non-specific symptoms that can be associated with PID are fever, abnormal uterine bleeding, urinary frequency, and abnormal vaginal discharge. All patients suspected of having PID should undergo a urine pregnancy test to rule out ectopic pregnancy, Nucleic Acid Amplification Test (NAAT) for *Chlamydia trachomatis/Neisseria gonorrhoeae* and *Mycoplasma genitalium*, Human Immunodeficiency Virus (HIV) screening, and microscopy of vaginal discharge. Findings that can support the diagnosis of PID include temperature > 101 Fahrenheit (F), abnormal cervical or vaginal mucopurulent discharge or cervical friability, presence of abundant white blood cells (WBCs) on saline microscopy of vaginal secretions (>15-20 white blood cells/high power field), documentation of anogenital tract infection with *N. gonorrhoeae, C. trachomatis, M. genitalium* [5]. However, negative test results does not rule out PID, and hence a presumptive clinical diagnosis of PID is made in sexually active young females presenting with pelvic or lower abdominal pain and satisfies one of the three minimum diagnostic criteria set forth by the Center for Disease Control and Prevention (CDC) including cervical motion tenderness, uterine tenderness, or adnexal tenderness on pelvic examination. Although the sensitivity of this clinical diagnosis is only 65-90% [6], empiric therapy should not be delayed due to the potential short and long-term consequences of untreated PID. Additional imaging is typically reserved for cases of diagnostic uncertainty or to assess for complications (e.g., tubo-ovarian abscess). Imaging studies commonly used are computed tomography (CT), magnetic resonance imaging (MRI), and pelvic ultrasound. Laparoscopy can provide a more accurate diagnosis of salpingitis, but it cannot detect endometritis or mild inflammation of the fallopian tubes, and given its invasive nature, it is typically not performed [7].

Treatment of PID consists of broad-spectrum antibiotics to target the polymicrobial nature of the infection, which often includes *Chlamydia trachomatis, N. gonorrhea*, and common anaerobic and aerobic bacteria. Empiric therapy is warranted without waiting for microbiological confirmation, given the potentially severe sequelae of untreated PID. Outpatient empiric treatment consists of intramuscular ceftriaxone 500 mg once (1 gram if weight >= 150 kg), oral doxycycline 100 mg twice daily, and oral metronidazole 500 mg twice daily for a 14-day course [8]. Hospitalization is indicated in the following scenarios: pregnancy, complicated by tubo-ovarian abscess, need to rule out surgical emergencies (e.g., appendicitis), severe illness, severe nausea or vomiting, high fever, or failure of outpatient antibiotics [9]. Inpatient empiric treatment consists of intravenous formulations of the aforementioned antibiotics and transitioning to oral antibiotics when there is clinical improvement over 24-48 hours. First line and alternative antibiotic tables reproduced from the 2021 Centers for Disease Control and Prevention (CDC) Sexually Transmitted Infections Treatment Guidelines are shown below in Tables 3-5 [9].

**Table 3:** Recommended intramuscular or oral regimens for pelvic inflammatory disease Reference no. [9] IM: Intramuscular, PO: per os (by mouth).

Recommended intramuscular or oral regimens for pelvic inflammatory disease			
Ceftriaxone 500 mg IM in a single dose (1 g for persons >= 150 kg) plus doxycycline 100 mg PO two times daily plus metronidazole 500 mg PO two times daily for 14 days			
Or			
Cefoxitin 2 g IM in a single dose administered concurrently with probenecid 1 g PO in a single dose plus doxycycline 100 mg PO two times daily plus metronidazole 500 mg PO two times daily for 14 days			
Or			
Other parenteral third-generation cephalosporin (e.g., ceftizoxime or cefotaxime) plus doxycycline 100 mg PO two times daily plus metronidazole 500 mg PO two times daily for 14 days			

**Table 4:** Recommended parenteral regimens for pelvic inflammatory disease Reference no. [9] IV: Intravenous, PO: per os (by mouth).

Recommended parenteral regimens for pelvic inflammatory disease			
Ceftriaxone 1 g IV every 24 hours plus doxycycline 100 mg PO or IV every 12 hours plus metronidazole 500 mg PO or IV every 12 hours			
Or			
Cefotetin 2 g IV every 12 hours plus doxycycline 100 mg PO or IV every 12 hours			
Or			
Cefoxitin 2 g IV every 6 hours plus doxycycline 100 mg PO or IV every 12 hours			

**Table 5:** Alternative parenteral regimens for pelvic inflammatory disease Reference no. [9] IV: Intravenous, PO: per os (by mouth).

Alternative parenteral regimens for pelvic inflammatory disease			
Ampicillin-sulbactam 3 g IV every 6 hours plus doxycycline 100 mg PO or IV every 12 hours			
Or			
Clindamycin 900 mg IV every 8 hours plus gentamicin loading dose IV or IM (2 mg/kg body weight), followed by a maintenance dose (1.5 mg/kg body weight) every 8 hours; single daily dosing (3-5 mg/kg body weight) can be substituted			

Most tubo-ovarian abscesses (TOA) are also treated medically, but surgical or minimally invasive consideration should be made for TOA > 8 mm in size [4], failure of antibiotic therapy, ruptured abscess, or suspected sepsis. The patient’s sexual contacts within 60 days of symptom onset should undergo screening for sexually transmitted infections (STIs) and be treated accordingly. Expedited partner therapy can be considered, especially with those lacking healthcare [10]. Patients with PID should be counseled to avoid sexual activity until completion of therapy, resolution of symptoms, and sexual partners have been evaluated and/or treated for potential STIs. All patients with PID, and especially those who are treated as an outpatient, should have close follow-up within 48-72 hours to ensure there is clinical improvement and evaluate for complications if there is no clinical improvement [11].

## Conclusions

Pelvic inflammatory disease is a common condition that is encountered in the outpatient and emergency room setting, which has no diagnostic gold standard, and so clinical diagnosis remains of utmost importance. Prompt diagnosis and treatment are crucial to reduce the risk of short- and long-term complications, including recurrence (5-25%), chronic pelvic pain, infertility, and ectopic pregnancy. PID can cause permanent injury to the fallopian tube, including loss of ciliary action, fibrosis, and occlusion, leading to hydrosalpinx, tubal infertility, and ectopic pregnancy. It should be noted that these long-term consequences can still occur despite appropriate antibiotic therapy.
